# Echocardiographic assessment of coronary microvascular dysfunction: Basic concepts, technical aspects, and clinical settings

**DOI:** 10.1111/echo.15059

**Published:** 2021-05-05

**Authors:** Andreina Carbone, Antonello D’Andrea, Simona Sperlongano, Ercole Tagliamonte, Giulia Elena Mandoli, Ciro Santoro, Vincenzo Evola, Francesco Bandera, Doralisa Morrone, Alessandro Malagoli, Flavio D’Ascenzi, Eduardo Bossone, Matteo Cameli, Echocardiography study group of the Italian Society of Cardiology

**Affiliations:** ^1^ Department of Cardiology University of Campania “Luigi Vanvitelli” Naples Italy; ^2^ Department of Cardiology and Intensive Coronary Unit “Umberto I” Hospital Nocera Inferiore (SA) Italy; ^3^ Department of Medical Biotechnologies Division of Cardiology University of Siena Siena Italy; ^4^ Department of Advanced Biomedical Sciences University of Naples “Federico II” Naples Italy; ^5^ Department of Clinical and Experimental Medicine University of Palermo Palermo Italy; ^6^ Heart Failure Unit Chair of Cardiology IRCCS Policlinico San Donato San Donato Milanese, Milan Italy; ^7^ Department of Biomedical Health Science University of Milan Milan Italy; ^8^ Cardiothoracic Department Cisanello Hospital University of Pisa Pisa Italy; ^9^ NOCSAE Hospital Modena Italy; ^10^ Division of Cardiology Cardarelli Hospital Naples Italy

**Keywords:** coronary flow reserve, coronary microvascular dysfunction, coronary physiology, Doppler echocardiography, microcirculation

## Abstract

Coronary flow reserve is the capacity of the coronary circulation to augment the blood flow in response an increase in myocardial metabolic demands and has a powerful prognostic significance in different clinical situations. It might assess with invasive and noninvasive technique. Transthoracic echocardiography Doppler is an emerging diagnostic technique, noninvasive, highly feasible, safe for patient and physician, without radiation, and able to detect macrovascular and microvascular anomalies in the coronary circulation. This review aims to describe the benefit and limits of echocardiographic assessment of coronary flow reserve.

## 1 INTRODUCTION

Coronary flow reserve (CFR), assessed by transthoracic echocardiography Doppler (TTE), is a noninvasive diagnostic technique able to reflect presence of macrovascular as well as microvascular disease in the coronary circulation, with the advantages of being highly feasible, safe for patient and physician, and not associated with any radiation.

It may represent a diagnostic tool for coronary microvascular dysfunction (CMD), while in the setting of ischemic cardiac disease is useful in identification and assessment of functional significance of coronary lesions as well as for the follow‐up of patients after coronary interventions. In addition, CFR has also showed a powerful prognostic significance in different clinical situations.

## 2 CFR AND MICROCIRCULATION: GENERAL CONCEPTS AND PHYSIOLOGY

Coronary circulation is characterized by complex morphology and physiology. Coronary arteries bifurcate into smaller vessels,^1^ divided into large arteries (diameter >500 µm) with capacitance function, small arteries, or prearterioles (diameter between 100 and 500 µm), that represent the intermediate compartment with a measurable pressure drop along their length and arterioles (diameter <100 µm).^1^ The coronary microcirculation includes vessels with diameters below ∼300 µm ranging from small arterioles to venules and is the site of regulation of flow resistance.^1^ The coronary microcirculation plays a key role in the myocardial perfusion.

Myocardial perfusion is predominantly diastolic with unique profile of coronary blood flow (CBF) velocity.^2^ Various mechanisms (metabolic, neurohumoral, and myogenic) intervene in the regulation of coronary flow in the prearteriolar and arteriolar microcirculation that allow stable flow across a large range of perfusion pressures.^3^ The myogenic autoregulation consists in the change of distal prearterioles diameter in response to flow changes and to increased pressure.^3^

The presence of functional and/or structural abnormalities of this circulatory pathway may impair the myocardial perfusion, a condition referred as *coronary microvascular dysfunction* (CMD). This term was referred to a large number of clinical scenarios characterized by evidence of a reduced CFR in the absence of obstructive epicardial disease.^4^ CMD includes any pathology that may damage the microvasculature, including endothelial dysfunction, coronary spasm, inflammation, and atherosclerosis.^5^

The coronary microvasculature cannot be directly visualized in vivo (vessels <300 µm), but the CMD may be assessed by many invasive and noninvasive technique, evaluating the CFR, which represents an integrated measure of CBF in both the macro‐ and microcirculation and consists in the ratio of hyperemic to baseline blood flow.^6^

In the absence of obstructive stenosis of the epicardial arteries, reduced CFR is a marker of CMD, but because obstructive disease of the epicardial arteries and CMD often coexist, discrimination between the effects of these two conditions on myocardial perfusion can be difficult. CFR is influenced by several factors: metabolic demand, diastolic time, systolic arterial blood pressure, heart rate, age, and sex.^7^

Pharmacological stressors are usually used to produce hyperemic response for CFR calculation. In clinical practice, the most used agents are dipyridamole and adenosine.^6^ Both inhibit phosphodiesterase, increasing cellular cyclic adenosine monophosphate (cAMP) and cyclic guanosine monophosphate (cGMP), with consequent coronary dilation.^8^ Coronary vasodilators are usually administered intravenously in noninvasive techniques, but adenosine is generally given intracoronary when measuring CFR in the catheterization laboratory. Cold pressor test is a nonpharmacologic stressor.^6^ It induces hyperemic vasodilation that depends totally on the endothelial release of nitric oxide, whereas both adenosine and dipyridamole stimuli are partially dependent on endothelial function.^9^ Cold pressor test is performed according to a standardized protocol, by placing the subject's hand and distal part of the forearm in ice‐water slurry for 3 minutes.^9^

## 3 CMD: PATHOGENESIS AND MECHANISMS

Multiple mechanisms are potentially responsible for CMD and can be classified in structural, functional, and extravascular changes.

Structural anomalies include vascular wall infiltration, vascular remodeling, perivascular fibrosis, and luminal obstruction. Smooth cell hypertrophy and the increased deposition of collagen, such as in hypertension or in hypertrophic cardiomyopathy (HCM), determine thickening of the media and intima tunica.^10^

Functional alterations are related to impaired dilatation or increased constriction of the coronary arterioles and prearterioles.^6^ Impaired vasodilatation can be due to a pathological endothelium‐dependent pathway (such as acetylcholine, and serotonin and flow‐mediated) or to endothelium‐independent pathways (eg, involving adenosine). On the other hand, increased vasoconstriction can be caused by increased levels of endothelin‐1, catecholamines, and acetylcholine.^10^

Extravascular anomalies consist in increased heart rate, with reduction in diastolic perfusion time, reduced driving blood pressure and extramural systolic compression, more severe in the endocardial layers.^11^

These different mechanisms frequently are simultaneously and can be observed in the absence and in the presence of myocardial disease and obstructive coronary artery disease (CAD).

## 4 CRF ASSESSMENT BY ECHOCARDIOGRAPHY: TECHNICAL ASPECTS

The left anterior descending (LAD) coronary CBF profile can be recorded with pulsed‐wave Doppler, either with transesophageal echocardiography (TEE), sampling the proximal tract, or TTE exploring the mid‐distal tract. Recently, the success rates to measure CBF of right coronary artery (RCA) and left coronary artery (LCA) have been increasing owing to the advancement in ultrasonic technology.^12^, ^13^ CBF velocity evaluated with Doppler is represented by a biphasic wave, with a lower peak during systole and a higher peak during diastole, for the effect of myocardial contraction.^14^ Several parameters might be measured from Doppler tracings of LAD artery flow, including systolic flows, time–velocity integrals, and mean flows, but the most used parameter is the peak diastolic flow: It is easy to measure and reproducible, and it has a closest correlation with CFR measured with positron emission tomography (PET).^15^ Second harmonic imaging and high frequency transducers (up to 8 MHz) provide better definition of coronary artery and improved resolution. Contrast agents also improved the signal‐to‐noise ratio, increasing the feasibility of transthoracic imaging of the LAD artery.^16^

### 4.1 Detection of coronary arteries

**FIGURE 1:**
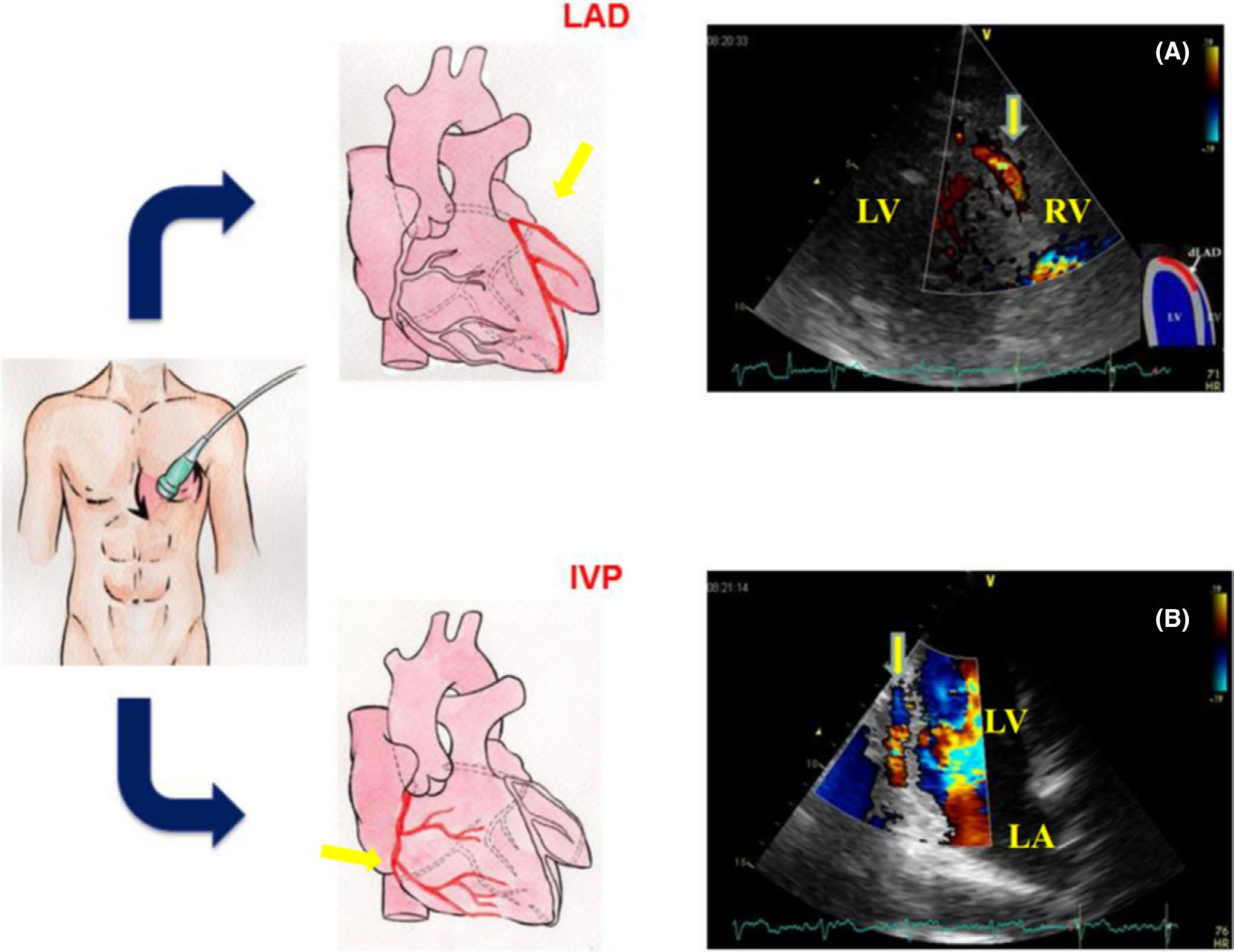
Color Doppler images of the distal left anterior descending coronary artery (LAD) and interventricular posterior coronary artery (IVP). The modified apical 4‐chamber (A) and 2‐chamber (B) positions, with cranial angulation of the transducer and a Nyquist limit of 20‐67 cm/s, allow optimal identification of the diastolic coronary flow. LV = left ventricle; RV = right ventricle; LA = left atrium

The principal coronary artery investigated is the LAD branch. It can be divided in three tracts: proximal, intermediate, and distal. The key reference points to detect the proximal tract are the left atrial appendage and the pulmonary artery. The intermediate tract key reference points are the septal perforans branches. The distal LAD tract is more suitable to investigate coronary microvascular function because it is between large epicardial arteries and microvasculature. This tract can be investigated in B‐mode and under Color Doppler guidance and by using growing delivery frequencies (5–7 MHz) in the second harmonic.^17^, ^18^ For a better visualization, the setting depth should be reduced approximately to 6–10 cm. The acoustic window is, in the left decubitus position, around the midclavicular line in the fourth or fifth intercostal space (Figure 1). The distal LAD can be assessed from the low left parasternal position to a modified apical five‐chamber position at varying levels using different short‐ and long‐axis views in the anterior interventricular groove before, at or after the apex of the left ventricle.^19^ The CBF in the distal LAD is searched for under the color Doppler flow. Angle correction is necessary in each examination. CBF is characterized by a biphasic flow pattern with a larger diastolic component and a smaller systolic one (Figure 1).

Posterior descending artery (PDA), usually the distal part of the RCA, can be assessed from fourth and fifth intercostal spaces in the apical long‐axis position in a modified two‐ or three‐chamber view with caudal tip of the transducer in the posterior interventricular groove^19^ (Figure 1). Unlike the LAD, the scanning depth for the RCA and LCA should be set at 12‐15 cm. It is possible to assess the RCA and LCA proximal and mid‐tract in a modified parasternal short axis focused on great vessels, but pulsed‐wave Doppler examination is rarely correct because the angle between the direction of CBF and Doppler beam exceeds 60 degrees. The first or second obtuse marginal branches presenting distal parts of the LCA can be assessed from fourth and fifth intercostal spaces in the apical long‐axis position in a modified four‐ or five‐chamber view at either lateral or inferior wall of the left ventricle.^19^

Feasibility of TTE in assessing coronary arteries with the addition of harmonics and contrast agents has been reported to be as high as 100% for distal LAD and 33%–97% for the PDA.^17^, ^18^

### 4.2 Pitfalls in detecting coronary arteries

There are many pitfalls regarding the CFR execution by TTE. First, regarding the assessment of coronary arteries, some branches of coronary arteries can occasionally be confused with the LAD. To avoid this mistake, the LAD should be visualized in the anterior interventricular groove in its entire length with the evidence of left main coronary artery (LMCA) and distal LAD. Furthermore, LAD can be confused with extracardiac arteries on color Doppler, such as the left internal thoracic artery (LITA), but, in this case, the pulsed‐wave Doppler shows a typical peripheral arterial flow with high systolic flow velocity. Larger cardiac veins can be distinguished for their three‐phasic, predominantly systolic, flow with respiratory variations and for their position, closer to the right ventricle.^20^

### 4.3 CBF velocity and CFR assessment with pulsed‐wave color Doppler

**FIGURE 2:**
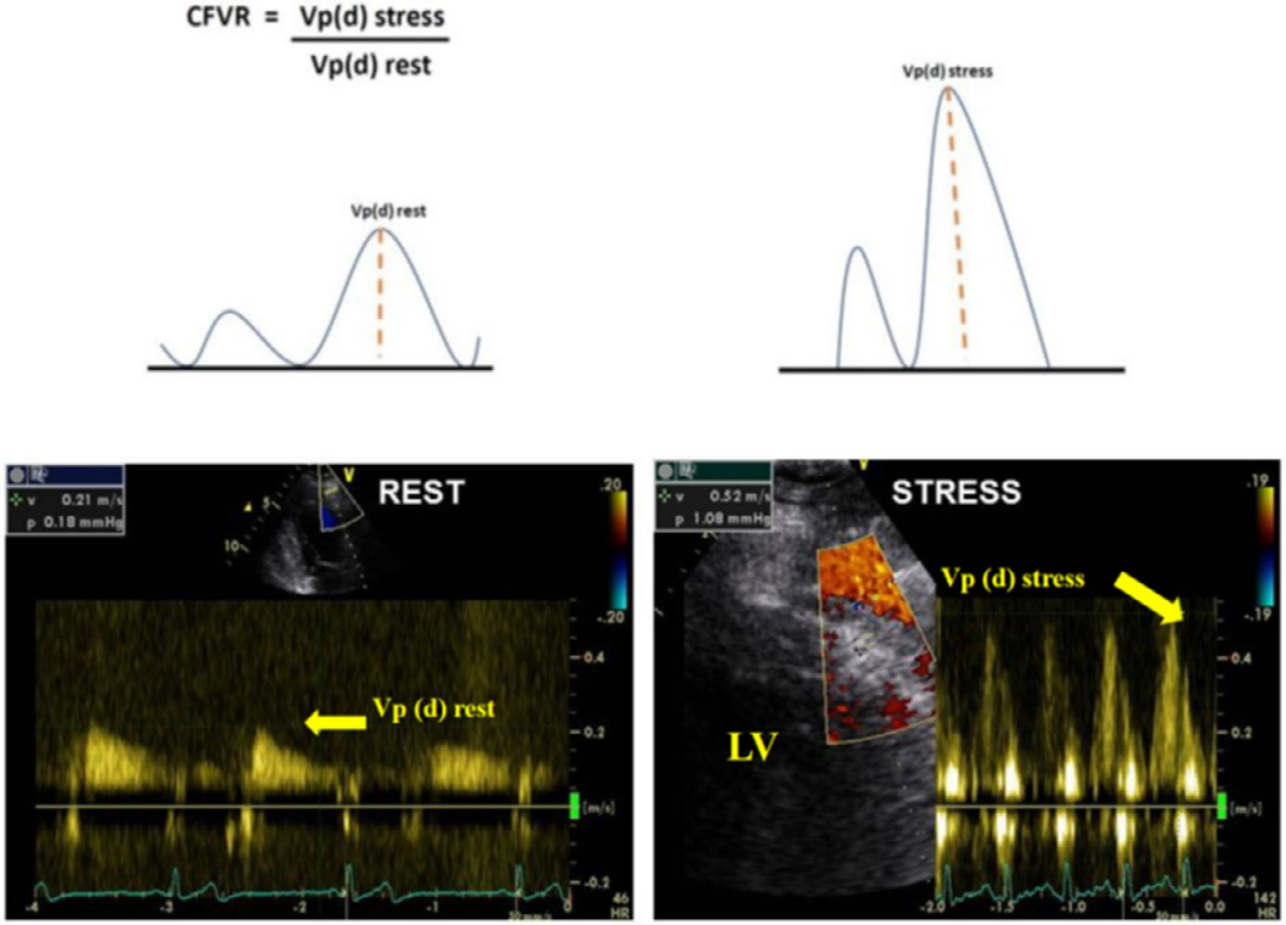
an example of normal coronary flow reserve in a healthy subject. Velocity patterns are registered by pulsed‐wave Doppler. Resting laminar peak diastolic velocity in normal coronary artery is from 0.21 ± 0.08 m/s to 0.28 ± 0.09 m/s, and the velocity will not exceed 1 m/s even in case of its three‐ fourfold increase in the stenosis site. CFR is expressed as the ratio of coronary flow velocity under maximal vasodilatation to coronary flow velocity at rest. In this case, the basal peak diastolic flow velocity is normal, and it increases more than 2 times after dipyridamole, and therefore, CFR is normal. LV = left ventricle; Vp(d) = velocity peak (diastolic); CFVR = coronary flow velocity ratio

To obtain a correct CBF velocity and CFR assessment, it is necessary that the correct Doppler angle and the sample volume are maintained in the correct position throughout the infusion of the vasodilator agent.^19^ The sample volume should be sized (~ 3.0 mm) and placed within the coronary artery.^21^ Time velocity integral, peak velocity, and mean velocity in systole and diastole should assessed at rest and during the pharmacological stimuli. The diastolic blood flow velocity is the most used parameter. Furthermore, it is possible to assess the diastolic and systolic flow length of time ^22^ (Figure 2). The normal value of the peak diastolic velocity in distal LAD was 21.2 ± 7.9 cm/s, and the duration of diastolic coronary artery flow was 58.5 ± 6.4% of the R‐R interval at rest and with normal heart rates (60–100 b/m), and also coronary flow velocities showed a nonsignificant decrease from proximal segments to distal segments.^23^

Adenosine protocol usually consists in intravenously infusion at the rate of 140 mcg/kg/min for 6 minutes, measuring CBF velocity before and after of adenosine infusion. Adenosine has a short half‐life, compared with dipyridamole (up to 30 minutes) and CFR achieved with adenosine can be obtained with dipyridamole infusion rate of 0.84 mg/kg for 6 minutes or in two separated doses, as suggested by the 2008 recommendations of the European Association of Echocardiography.^24^ Table 1 summarized the stress echocardiography protocols for CFR assessment.

**TABLE 1:** Dipyridamole and adenosine stress echocardiography protocol

General methodology during stress echo	Perform 12 lead ECG in resting condition and each minute throughout examination. An ECG is continuously displayed on echo monitor. Cuff blood pression is measured in resting condition and at each stage.
Contraindications	Active bronchospasm. > or equal 2nd degree AV block. SBP <90 mm Hg. Use of methylxanthine. Remote history of reactive airway disease Chronic dipyridamole therapy, recent (<12 h) coffee, tea, chocolate ingestion. Avoid dipyridamole stress echocardiography in patients presenting severe bilateral carotid disease with unknown Willis polygon circulation, due to the potential risk of brain ischemia.
Echocardiography imaging acquisition	Assess the distal LAD artery and the CBF velocity (peak velocity) at rest and after starting dipyridamole/adenosine infusion. If possible, RCA and LCA can be visualized. Perform regional wall motion analysis at rest and at peak. Echocardiography is continued monitored and intermittently stored.
Standard dipyridamole protocol	Dipyridamole intravenous infusion of 0.84 mg/kg over 10 min in two separate infusions: 0.56 mg/kg over 4 min (“standard dose”), followed by 4 min of no dose and if still negative, an additional 0.28 mg/kg over 2 min. Atropine (doses of 0.25 mg up to 1 mg) can be administered
Rapid dipyridamole protocol	Dipyridamole 0.84 mg/Kg can be given over 6 min
Adenosine protocol	Adenosine can be infused at maximum dose of 140 µg/kg/min over 6 min
After stress	Aminophylline (240 mg iv) should be available for immediate use in case of an adverse event occurs and routinely used at the end of the test

Abbreviations: AV = atrio‐ventricular; CBF = coronary blood flow; ECG = electrocardiogram; LAD = left anterior descending; LCA = left coronary artery; RCA = right coronary artery; SBP = systolic blood pressure.

After measuring the baseline and the hyperemic coronary flow velocities, CFR should be calculated as the ratio of CBF velocity under maximal vasodilatation to the same parameter at rest.^25^ Many authors assume that the peak velocity (VpD) can be used for CFR evaluation and the cutoff <2 for predicts significant LAD stenosis in distal LAD and in PDA,^26^, ^27^ whereas CFR <1 denotes LAD or PDA occlusion.^28^, ^29^

In patient with CAD, CFR is related to the severity of coronary disease, while in case of angiographically normal arteries it is a marker of microvascular dysfunction.^30^

Recently, the ABCDE protocol, validated by the Stress Echo 2020 study group of the Italian Society of Echocardiography and Cardiovascular Imaging, has been proposed during stress echocardiography to investigate dyspnea of cardiac origin, analyzing 5 parameters: regional wall motion (step A); pulmonary congestion with B‐lines (step B); LV contractile reserve (step C); coronary microvascular dysfunction with CFR (step D); and EKG‐based heart rate reserve during exercise (step E).^31^ Reduced CFR is often accompanied by regional wall motion anomalies, abnormal left ventricle contractile reserve, and pulmonary congestion during stress and shows independent value over segmental motion anomalies in predicting an adverse outcome.^32^

## 5 CFR ASSESSED BY TEE

CBF velocity can be assessed by TEE with Doppler examination of the proximal LAD coronary artery. This is a simple, reproducible, safe, and reliable method for coronary flow reserve assessment and is correlated with CBF velocity derived from intracoronary Doppler guide wire studies.^33^ Coronary artery imaging was performed after routine cardiac examination. The LMCA was visualized, with the probe approximately 28 cm from the mouth, at a level above the aortic leaflets.^34^ The LMCA, arising from the corresponding sinus, is seen as an echo‐free structure and small adjustments in transducer orientation are necessary to visualize the artery, its bifurcation, and LAD. CBF velocity was evaluated by pulsed‐wave Doppler of the ostial part of the LAD.^34^

## 6 MYOCARDIAL CONTRAST ECHOCARDIOGRAPHY (MCE)

Quantitative contrast echocardiography has been shown to be useful for assessing global and regional CFR using different pharmacologic stimuli. This technique seems feasible for the assessment of mechanistic insights at coronary microcirculation.^35^ MCE utilizes gas‐filled microbubbles to produce myocardial opacification on ultrasound examination. After complete destruction of microbubbles using a beam of high‐intensity ultrasounds, the replenishment of myocardial microcirculation may be assessed as a time‐intensity curve, with a mathematical function.^36^ In patients without significant stenosis of coronary arteries, contrast infusion determines homogeneous opacification of the myocardial wall, because microbubbles continuously replaced those that are destroyed by ultrasounds. In case of microvascular defects, CBF increases during pharmacological/physical stress and determines regional differences of opacification. An excellent correlation was found with PET.^36^ MCE did not receive regulatory authorities approval for clinical use, and the technique remains an option for research.^36^

## 7 THE PROGNOSTIC ROLE OF CFR IN DIFFERENT CLINICAL SETTINGS

Cardiac microvascular function may be altered in many clinical conditions. CFR assessment has an important prognostic value and adds incremental information over the value of inducible wall motion abnormalities. Indeed, CMD evaluated by TTE is independent predictor of cardiovascular events in patients with suspected CAD. In a study by Li‐Ming Gan, 371 patients underwent to myocardial perfusion scintigraphy for suspected myocardial ischemia. The major cardiovascular events rate was 7.5% in patients without myocardial ischemia and normal CFR, whereas event rate was 24.2% in patients without ischemia but with reduced CFR, and 46.5% in patients with both myocardial perfusion scintigraphy–detected myocardial ischemia and a reduced CFR (*P* < .001).^37^ To better define microvascular dysfunction, Coronary Vasomotion Disorders International Study Group (COVADIS) created a consensus document describing invasive and noninvasive methods for detecting endothelial‐dependent and endothelial independent CMD,^4^ for the diagnosis of microvascular angina. The following criteria were proposed: (1) presence of symptoms suggestive of myocardial ischemia; (2) objective documentation of myocardial ischemia, as assessed by currently available techniques; (3) absence of obstructive CAD (<50% coronary diameter reduction and/or fractional flow reserve (FFR) > 0.80); and (4) confirmation of a reduced coronary blood flow reserve and/or inducible microvascular spasm.

## 8 CMD IN HYPERTENSION, DIABETES MELLITUS, AND SMOKING

Coronary microvascular function is compromised in patients with hypertension and/or diabetes. Hypertension impairs endothelial function and increases microvascular resistance.^38^ CFR was impaired in hypertensive patients compared with healthy subjects, also in patients with prehypertension.^39^ The impairment of CFR occurs very early in hypertension, before organ damage is evident.^39^ Histological studies have showed increased arteriolar media area and interstitial fibrosis in patients with arterial hypertension and angina pectoris in the absence of significant CAD.^40^

CMD has been reported in patients with diabetes mellitus and normal coronary arteries.^41^ Hyperglycemia and oxidative stress determine accumulation of advanced glycation end products, with consequent increased interstitial fibrosis.^42^ Consequently, diabetic patients have a reduced CFR, as demonstrated with different techniques and CFR <2 is an independent predictor of death and nonfatal myocardial infarction.^43^

Tobacco smoking is associated with epicardial atherosclerosis and impaired endothelial function. It was reported that CRF was lower in smokers than in controls. CFR decreases, with no change in hemodynamic parameters, after acute smoking, determined invasively ^44^ and using PET.^45^

## 9 CMD IN VALVULAR HEART DISEASE

Patients with moderate and severe aortic stenosis have shown an impairment of CFR.^46^ Marko et al have shown that CFR <1.85 has high sensitivity and specificity in predicting adverse outcome during long term follow‐up.^47^ The resolution of stenosis with transcatheter aortic valve implantation (TAVI) determines a decrease of microvascular resistance and an improvement in coronary vasodilatory reserve.^48^

CFR, assessed by dipyridamole‐induced coronary vasodilation, is greatly impaired in patients with aortic regurgitation, left ventricle hypertrophy, exertional chest pain, and normal coronary arteries.^49^ CFR is also impaired in patients with severe mitral regurgitation and improves after successful mitral surgery for the reduction of left ventricular preload, volume, and hypertrophy.^50^

### 9.1 CMD in cardiomyopathies

**FIGURE 3:**
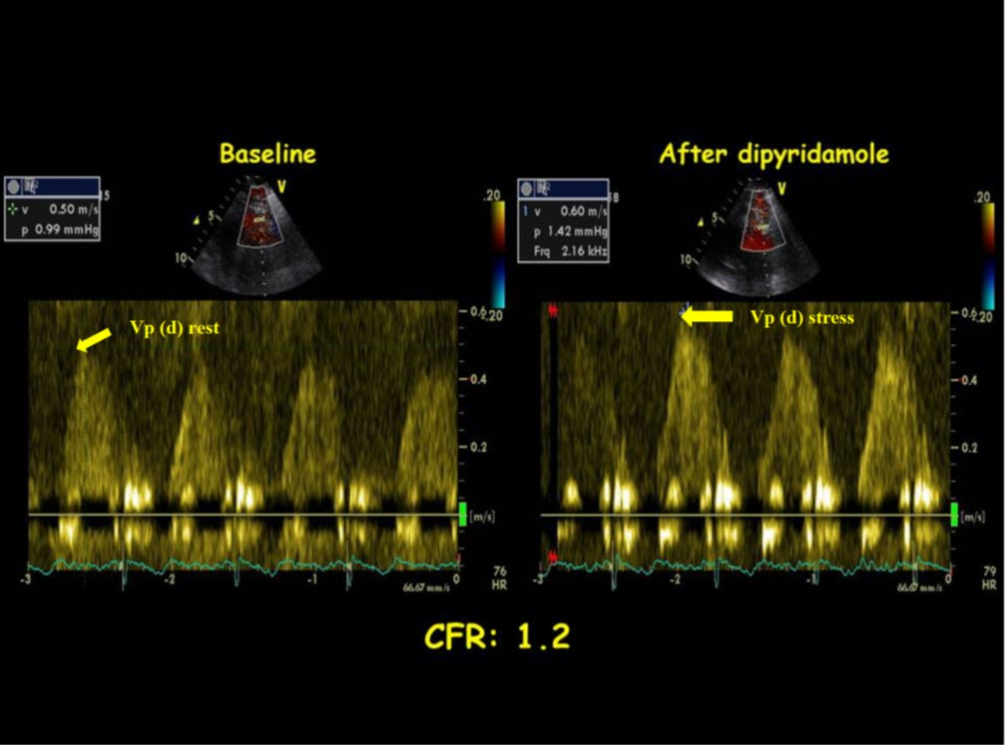
impaired CFR in an HCM patient with normal epicardial coronary arteries. Basal increased coronary flow with high peak diastolic flow velocity in distal LAD at rest; after dipyridamole, the increase of peak diastolic flow velocity is slight, and CFR is equal to 1.2. Vp(d)= velocity peak (diastolic)

Reduction in CFR is a recognized feature in HCM and is a strong predictor for future cardiovascular events.^51^ Patients with HCM show important wall thickening of coronary arterioles, due to intimal hyperplasia and interstitial fibrosis reduces capillary density, with angina, progressive deterioration of cardiac function ^52^ (Figure 3). Severe CMD is situated in the hypertrophied septum, but also in the nonhypertrophied left ventricle free wall using PET ^53^ and CMR.^54^ Severe impairment of microvascular function is more frequent in HCM patient with sarcomere mutations.^55^ CFR was lower in patients with obstructive HCM compared with nonobstructive HCM,^56^ and alcohol septal ablation results in improvement of CFR.^57^

CFR is decreased in patients with nonischemic dilated cardiomyopathy.^58^ An impaired CFR identifies patients at higher risk of, such as death and worsening of clinical conditions.^59^

### 9.2 CMD in heart transplant

Cardiac allograft vasculopathy (CAV) is characterized by diffuse concentric myo‐intimal thickening of the distal epicardial artery and the small endocardial vessels.^60^ Annual coronary angiography is the most common approach to evaluate the progression of CAV. Recently, the assessment of CFR on TTE has been proposed as an alternative method to diagnosis CAV. A recent study has shown that CFR is sensitive to assess microvascular and macrovascular dysfunction, and when CFR is combined with dobutamine stress echocardiography, CAV can be accurately diagnosed with high specificity.^61^

### 9.3 Chronic systemic inflammation and microvascular dysfunction

Patients with autoimmune diseases, such as rheumatoid arthritis and systemic lupus erythematosus, have increased cardiovascular morbidity and mortality for premature coronary artery disease.^62^ Systemic inflammation damages the coronary microcirculation. Leucocytes and soluble factors play an important role in accelerating vessel atherosclerosis. These patients have impaired CFR without significant coronary disease, and the impairment is correlated with the duration of the disease.^63^

## 10 LIMITATIONS

CFR measured with Doppler has some limitations. Echocardiography is operator dependent and demonstrates considerable intra‐observer and inter‐observer variability and can be hindered by artifacts, particularly in obesity and lung disease. CFR assessment inter‐observer variability is acceptable (coefficient of variability <10%), as reported previously.^64^, ^65^ The CBF is not calculated directly, because it is not possible to measure accurately the diameter of the vessel; CFR is calculated without considering the change of coronary artery diameter during drug infusion. The changes in coronary diameter during vasodilator infusion introduce a source of error.^66^

This method correlates well with flow acquired from an intracoronary Doppler wire but is poorly correlated with myocardial perfusion reserve calculated by PET.^3^ Some side effects (hypotension and/or bradycardia, headache, dizziness, and/or nausea) preclude maximal pharmacological stress in less than 5% of patients.^67^

## 11 CONCLUSIONS

TTE, a noninvasive and widely used method, can be used for the diagnosis of CMD.

After an adequate period of training, detection and measurement of distal LAD and RCA flow and CFR by TTE is feasible in more than 90% of patients. Noninvasive serial measurements of coronary flow velocity at rest and after stress are useful for understanding the physiology and pathophysiology of microcirculation, for diagnosis and the follow‐up of different clinical conditions.

## ETHICAL APPROVAL

The protocols of studies cited in this review, reporting the results of human experimentation, were approved by local institutional review board (IRB), and informed consents for the studies were obtained from all human subjects in accordance with the WORLD Medical Association Declaration of Helsinki: Ethical principles for medical research involving human subjects, 2013.

The figures in our review are original, not published previously and that subjects granted permission for the use of their data/images in published articles.

## Data Availability

Data sharing not applicable to this article as no datasets were generated or analyzed during the current study. The data that support the findings of this study are openly available.
